# Engineered protein A ligands, derived from a histidine-scanning library, facilitate the affinity purification of IgG under mild acidic conditions

**DOI:** 10.1186/1754-1611-8-15

**Published:** 2014-07-01

**Authors:** Masayuki Tsukamoto, Hideki Watanabe, Ayako Ooishi, Shinya Honda

**Affiliations:** 1 Department of Medical Genome Sciences, Graduate School of Frontier Sciences, The University of Tokyo, Kashiwa, Japan; 2 Biomedical Research Institute National Institute of Advanced Industrial Science and Technology (AIST), Higashi, Tsukuba, Ibaraki 305-8566, Japan; 3 Manufacturing Technology Association of Biologics, Chuo-ku, Kobe, Japan

**Keywords:** Antibody, Affinity purification, *Staphylococcal* protein A, Histidine-scanning Library, Combinatorial screening

## Abstract

**Background:**

In antibody purification processes, the acidic buffer commonly used to elute the bound antibodies during conventional affinity chromatograph, can damage the antibody. Herein we describe the development of several types of affinity ligands which enable the purification of antibodies under much milder conditions.

**Results:**

*Staphylococcal* protein A variants were engineered by using both structure-based design and combinatorial screening methods. The frequency of amino acid residue substitutions was statistically analyzed using the sequences isolated from a histidine-scanning library screening. The positions where the frequency of occurrence of a histidine residue was more than 70% were thought to be effective histidine-mutation sites. Consequently, we identified PAB variants with a D36H mutation whose binding of IgG was highly sensitive to pH change.

**Conclusion:**

The affinity column elution chromatograms demonstrated that antibodies could be eluted at a higher pH (∆pH**≧2.0) than ever reported (*∆pH* = 1.4) when the *Staphylococcal* protein A variants developed in this study were used as affinity ligands. The interactions between *Staphylococcal* protein A and IgG-Fab were shown to be important for the behavior of IgG bound on a SpA affinity column, and alterations in the affinity of the ligands for IgG-Fab clearly affected the conditions for eluting the bound IgG. Thus, a histidine-scanning library combined with a structure-based design was shown to be effective in engineering novel pH-sensitive proteins.

## Background

Monoclonal antibodies are currently one of the most important applications in biotechnology and as a promising class of drugs in the biopharmaceutical industry [1,2]. Affinity chromatography is commonly used for antibody purification on both the laboratory and industrial scale because it provides easy, fast and selective purification [3]. However, conditions for efficiently eluting monoclonal antibodies from affinity columns require improvement. Conventional affinity chromatography for antibodies uses an acidic buffer solution for elution, which would cause antibodies to denature and/or to form aggregates, leading to immunogenicity and other adverse reactions [4,5]. To solve these problems, several types of affinity ligands have been developed for the elution of antibodies under milder conditions [6-11].

The affinity ligands commonly used for antibody purification are *Staphylococcal* protein A (SpA) and *Streptococcal* protein G (SpG). Optimizing the pH-sensitive interactions of these affinity ligands with the target antibodies could improve elution conditions and guarantee the quality of purified antibody. A number of studies using various strategies [7,9-19] to modulate the pH sensitivity of protein-protein interactions have been carried out. Most of pH-sensitive* proteins in these studies were produced by introducing histidine-mutations based on structural designs, such as that by Brown *et al.*[7] on engineered pH-sensitive SpA B-domain (PAB) variants. In previous studies, we developed rational strategies for histidine-mutations to produce pH-sensitive IgG-binding SpG [9] and SpA [11].

To obtain pH-sensitive proteins, it is important to explore the most effective positions for histidine-substitution. Gera *et al.*[10] performed a histidine-scanning mutagenesis study where a number of Sso7d protein variants with individual single histidine-substitutions were systematically examined; they identified a pH-sensitive mutant containing a single histidine substitution. In addition to these structure-based designs, combinatorial screening is also a promising approach. Murtaugh *et al.*[19] reported the engineering of pH-sensitive proteins using a histidine-scanning library of anti-RNase A single domain VHH antibodies, and identified pH-sensitive antibodies with multiple histidine substitutions that retained near wild-type affinity but with high sensitivity to pH change for elution.

In this paper, we describe the design and generation of pH-sensitive PAB variants by introducing a histidine-mutation using both structure-based design and combinatorial screening. First, a histidine-scanning library for PAB was created based on structural information. The effective histidine-mutation positions were evaluated from statistical analysis of the frequency of occurrence of amino acids in the sequences isolated using a T7 phage display screening. We also investigated the pH value at which IgG peaks eluted from PAB variant-immobilized affinity columns. To better understand the effects of histidine-mutation at molecular level, we evaluated the structural stability of each histidine-substituted PAB variant and its binding affinity to IgG by Surface Plasmon Resonance (SPR) analysis.

## Results and discussion

### Generation of histidine-scanning library

To design the histidine-scanning library, we focused on the IgG-Fc binding interface of PAB for the mutation positions to be randomized. Our previous study [9] revealed that positions near positively charged residues on a target protein were effective sites for histidine-mutation. First, positively charged residues (histidine, lysine and arginine) on IgG1-Fc under acidic conditions were searched using the structure of PAB complexed with IgG1-Fc [pdb: 1FC2] [20]. Distances from the positively charged residues in IgG1-Fc to all the PAB residues were calculated (Table 1). Next, the solvent exposure of each residue in PAB was calculated in order to select the residues on the protein surface using the subunit structure of PAB in the complex (Table 1).

**Table 1:** Mutation positions for histidine-scanning library

Number	5	6	9	10	11	13	14	15	17	24	25	27	28	31	32	35	36
Aminoacid residue of wild type	F	N	Q	Q	N	F	Y	E	L	E	E	R	N	I	Q	K	D
Distance (Å)	5.7	11	6.4	4.2	6.5	3.8	3.4	7.9	4.0	8.4	8.7	8.6	6.9	5.8	8.1	4.5	9.8
fSASA (%)	78	94	36	47	64	40	63	50	46	78	96	23	71	17	60	64	68
Mixed codon used in the library	YWT	MAY	CAW	CAW	MAY	YWT	YAT	SAW	CWK	SAW	SAW	CRY	MAY	MWT	CAW	MAW	SAT
Substituted amino acids in the library	H					H		H	H	H	H			H		H	
	Y	H	H	H	H	Y	H	D	Q	D	D	H	H	N	H	N	H
	L					L		Q		Q	Q			L		Q	

Distance in the table indicates the length between the PAB residue and the nearest positively charged residue in IgG-Fc. fSASA indicates the extent of solvent exposure of the side chain in wild-type amino acid residues of PAB. The seventeen mutation positions finally selected were classified into two groups: (1) the PAB residues were located within 9 Å from the positively charged IgG-Fc residues and (2) the PAB residues were located between 9 and 11 Å from the IgG-Fc residues, and with a fractional solvent-accessible surface area (fSASA) ((fSASA) = (SASA) (native)/SASA (denatured)) value of more than 68%. The following abbreviations are used for mixed bases: R = (A or G), Y = (C or T), M = (A or C), K = (G or T), S = (G or C) and W = (A or T). Substituted amino acid residues in the library indicate non-wild-type residues encoded by a mixed codon.

Several residues important in the interaction between PAB and IgG1-Fc have been previously identified by mutational experiments [7,11,21-24] and by molecular simulation calculations [25,26]. However, we did not take these positions into particular consideration for our library design in order to test a wide range of mutation positions in PAB.

The theoretical molecular diversity of a histidine-scanning library can be controlled by the number of mutation positions. In order to restrict the library to fit within the size range of T7 phage-display screening, we determined the maximum number of mutation positions. The seventeen mutation positions were finally selected (Table 1).

### Screening of histidine-scanning library

The histidine-scanning library was screened using a T7 phage-display method. The initial library size (5.4 × 10^7^ pfu) of T7 phages estimated by plaque assays was larger than the theoretical value (2.5 × 10^7^ pfu) of the objective library, so a highly diverse library was produced.

After each round of selection, the T7 phages were cloned and several dozen of the sequences were determined. The ratios of unique sequences out of the sequences determined after each round were as follows (the number of unique sequences/the number of total sequences determined): 100% (the initial library: 84/84), 100% (after the 1st round: 50/50), 97.5% (after the 2nd round: 78/84), 51.7% (after the 3rd round: 30/58), 25.0% (after the 4th round: 20/88), and 19.3% (after the 5th round: 18/93). Sixty one out of 93 clones examined after the final round had identical sequences (Additional file 1: Table S2). It should be mentioned that the frequency of occurrence of the wild-type or histidine residue at each mutation position fluctuated (Additional file 2: Figure S1, Additional file 3: Figure S2 and Additional file 4: Figure S3).

### Frequency of occurrence of amino acid residues of isolated PAB variants

**Figure 1:**
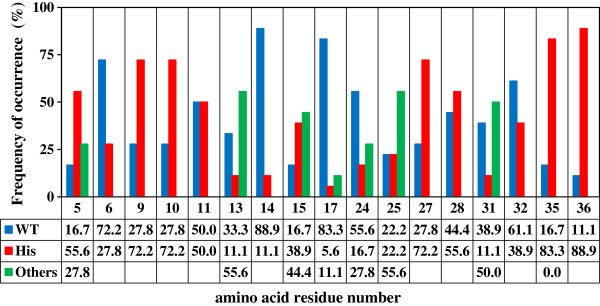
**Frequency of occurrence of amino acid residues after the final round.** The frequency of occurrence of amino acid residues was calculated from 18 unique determined sequences of PAB variants after the final round. The ordinate indicates the percentage of the frequency of occurrence. The abscissa indicates the amino acid residue number. WT, wild-type residue: His, histidine residue: Others, non-wild-type and non–histidine residues. The number in the lower three rows indicate percentage.

The frequency of occurrenc of the amino acids was calculated from 18 unique sequences determined from the final round (Figure 1). The positions at which the frequency of the histidine residue was less than 20% were: F13, Y14, L17, E24 and I31, which were previously shown to be essential for the interaction with IgG-Fc [6,7,21-26]. For example, Brown *et al.*[7] used ELISA to show that the binding affinity of rabbit IgG with a PAB mutant (F13H) at pH 8.0 was more than 100-fold weaker than with wild-type PAB.

The positions at which the frequency of occurrence of a histidine residue was more than 70% were expected to be effective histidine-mutation sites. They were: Q9, Q10, R27, K35 and D36. The frequency of a histidine residue at D36 was the highest, although D36 has been little investigated in previous studies [7,21-26]. Hence, a single histidine-substituted (D36H) PAB variant and four double histidine-substituted PAB ones containing the D36H mutation and either the Q9H, Q10H, R27H or K35H mutation were produced.

### Elution profile of bound IgG from the PAB variant columns

The affinity chromatograms with the PAB variants described above indicated that the pH values at which IgG eluted from all the PAB variant columns were higher than the elution pH from the PAB01 (Wild-type (WT)) column (Table 2, Additional file 5: Figure S4). First, the pH sensitivity of PAB02 (D36H) was clearly higher. For the double substitution PAB variants, the pH values of the IgG elution peaks from the PAB03 (Q9H, D36H) and PAB04 (Q10H, D36H) columns were significantly higher than that of the PAB02 (D36H) column. On the other hand, the pH required to elute IgG from the PAB05 (R27H, D36H) and PAB06 (K35H, D36H) column was similar to that of the PAB02 column. Therefore, the Q9, Q10 and D36 histidine-mutations showed much better pH sensitivity. The *∆pH* of the IgG elution peaks from the PAB03 and PAB04 columns was more than ever reported (Table 2, Additional file 6: Table S3).

**Table 2:** The pH values of the IgG elution peaks and the thermal stability of PAB variants

**Protein**	**Elution Peak**	***∆pH***	**Stability**	***∆T***_**m**_
	**pH**		***T***_**m **_**(K)**	
PAB01 (Wild Type)	3.5	-	346.4	-
PAB02 (D36H)	4.6	1.1	345.8	−0.6
PAB03 (Q09H, D36H)	5.5	2.0	343.7	−2.7
PAB04 (Q10H, D36H)	6.8	3.3	343.1	−3.3
PAB05 (R27H, D36H)	4.7	1.2	316.2	−30.2
PAB06 (K35H, D36H)	4.4	0.9	342.5	−3.9
PAB07 (Q09H)	3.9	0.4	344.9	−1.5
PAB08 (Q10H)	3.7	0.2	343.6	−2.8
PAB10 (Q32H)	4.2	0.7	341.4	−5.0

Affinity columns were prepared using the PAB variants. The captured IgG on each column was eluted with a decreasing pH gradient. The pH value in the table indicates the peak position of the eluted IgG on the affinity chromatography. The midpoint of the transition during thermal denaturation (*T*_m_) was determined from circular dichroism melting measurements.

*∆pH* = (the pH value at which IgG eluted from mutant column) - (the pH value at which IgG eluted from wild-type column).

*∆T*_m_ = (the thermal stability of PAB mutant expressed as *T*_m_) - (that of the thermal stability of PAB wild-type, PAB01).

Next, we evaluated the effects of combinations of the histidine-mutations. The pH values for eluting IgG from a single histidine-substituted PAB variant (PAB07 (Q9H) or PAB08 (Q10H)) column was slightly higher than that required with PAB01. Thus, the large effects of Q9H and Q10H on the pH sensitivity of the PAB03 and PAB04 columns were likely due to the combination effect with D36H.

### Structural stability of PAB variants

We attempted to evaluate the effect of introducing a histidine-mutation on the structure and thermal stability of PAB variants. The circular dichroism (CD) spectra of all PAB variants at room temperature were similar to that of PAB01 (WT). The midpoint of the transition during thermal denaturation (*T*_m_) was determined from CD melting measurements (Table 2, Additional file 7: Figure S5). The pH-sensitive PAB variants with effective histidine-mutations (Q9H, Q10H and D36H) showed a slight decrease in thermal stability. An increase in the number of histidine-mutations led to a decrease in the *T*_m_ value. Thus, histidine-mutations apparently have additive effects on thermal stability. In summary, however, the decrease in thermal stability due to histidine-mutations was rather limited and would not prevent utilization of these mutants as affinity ligands.

### Binding assay of PAB variants

#### Interaction under neutral conditions

We examined the binding affinity of IgG for each of the four single histidine-substituted PAB variants under neutral conditions (pH = 7.4) (Table 3). Of these, PAB08 (Q10H) showed the weakest binding affinity, about 10-fold weaker than that of PAB01 (WT). The binding affinities of IgG for the double histidine-substituted PAB variants containing D36H were lower than those of their single substitution PAB variant counterparts. PAB03 (Q9H, D36H) was exhibited 56-fold weaker binding affinity than PAB01. Thus, the IgG binding of PAB variant columns in this study was decreased (Additional file 8: Figure S6). Still, except for PAB04, the interaction between IgG and each of the PAB variants with single or double histidine substitutions would be strong enough to retain IgG on an affinity column using these variants as ligands.

**Table 3:** Binding affinity of IgG for PAB variants

	**pH = 7.4**		**pH = 5.0**	
**Protein**	***K***_**D **_**(×10**^**−9**^**) (M**^**−1**^**)**	***K***_**D **_**ratio**	***K***_**D **_**(×10**^**−9**^**) (M**^**−1**^**)**	***K***_**D **_**ratio**
PAB01 (WT)	5.1 ± 3.2	-	7.6 ± 4.3	-
PAB02 (D36H)	33.6 ± 19.2	6.6	832 ± 443	110
PAB03 (Q9H, D36H)	288 ± 146	56	ND	-
PAB04 (Q10H, D36H)	6830 ± 3600	1300	ND	-
PAB05 (R27H, D36H)	80.7	16	2090	270
PAB06 (K35H, D36H)	550 ± 143	110	3860	510
PAB07 (Q9H)	13.9 ± 6.3	2.7	30.6	4
PAB08 (Q10H)	50.8 ± 20.5	10	951	120
PAB09 (R27H)	5.6 ± 1.3	1.1	7.0	0.92
PAB10 (Q32H)	2.8 ± 1.3	0.55	96.8	13

The binding assay was performed under neutral (pH = 7.4) and acidic (pH = 5.0) conditions using SPR. The IgG was immobilized on the surface of a CM5 sensor chip (GE Healthcare).

*K*_D_, equilibrium dissociation constant: *K*_D_ ratio, (*K*_D_ of histidine-substituted PAB variant)/(*K*_D_ of Wild-type PAB): ND, not determined.

#### Interaction under acidic conditions

The binding affinity of IgG for PAB01 under the acidic condition (pH = 5.0) (Table 3) was about 1.5-fold weaker than at neutral pH, still quite strong. PAB07 (Q9H) and PAB09 (R27H) also retained strong affinity. Brown *et al.*[7] showed that the K35H PAB variant exhibited strong affinity under both neutral (pH = 8.0) and acidic (pH = 5.0) conditions.

On the other hand, the binding affinity of IgG for PAB02 (D36H) and PAB08 (Q10H) was more than 100-fold weaker than that for PAB01. The binding affinities for PAB03 (Q9H, D36H) and PAB04 (Q10H, D36H) were too weak to be determined under the acidic condition. Thus, histidine-mutations at Q9, Q10 and D36, as well as the double-mutations, Q9H plus D36H and Q10H plus D36H, appeared to have favorable pH-sensitivity. It should also be noted that histidine-mutation at D36 improved the pH sensitivity of all the four histidine-mutations under both neutral and acidic conditions.

#### The effects of histidine-mutations

The observed effects of the histidine-mutations could be explained based on crystal structure and biophysical data. Q9 and Q10 of PAB probably contribute to the interaction with IgG-Fc. The binding affinity of PAB08 (Q10H) under the neutral condition was about 10-fold weaker than that of PAB01 (WT). Five residues in IgG-Fc are found within 4 Å of Q10 of PAB. Therefore, the steric hindrance generated by the Q10H mutation likely affects the interaction with IgG-Fc. IgG elution from the affinity columns and SPR measurement results under the acidic condition demonstrated that the effects of histidine-mutation at Q10 are more significant than mutation at Q9. Because the positively charged residue (His435) in IgG-Fc resided near Q10 of PAB (<4.2 Å), electrostatic repulsion would increase when the pH value changes from neutral to acidic.

As described below, however, the decrease in binding affinity due to the D36H mutation (PAB02) was unlikely to be affected by modulation of the interaction of the IgG-Fc binding interface of PAB.

### Effect of the D36H mutation

Ile253 of IgG-Fc resides 6.89 Å from D36 of PAB in the PAB-IgG1-Fc complex [20]. Since this is not a short distance, the effects of the histidine-mutation at D36 obtained from the screening experiment are difficult to explain if the effect were caused by modulation of the interaction between PAB and IgG-Fc. In fact, it was previously observed that SpA binds not only to IgG-Fc but also to IgG-Fab [27-30]. We focused on the IgG-Fab binding interface of PAB.

We evaluated the binding affinity of IgG-Fc and IgG-Fab for PAB01 (WT) and PAB02 (D36H) (Tables 3 and 4) using SPR. The interaction between PAB02 and IgG-Fc was found to be similar to that of PAB01 under both neutral and acidic conditions. On the other hand, the binding affinity of PAB02 for IgG-Fab under the neutral condition was 2.3-fold weaker than that of PAB01, and the binding affinity of PAB02 under the acidic condition was too weak to be determined. These results suggested that the interaction of IgG-Fab with PAB would significantly contribute to the effects of histidine-mutation at D36.

**Table 4:** Binding affinity of IgG-Fc (upper rows) or IgG-Fab (lower rows) for PAB01 (WT) and PAB02 (D36H)

**Protein**	**pH = 7.4**		**pH = 5.0**	
	***K***_**D **_**(×10**^**−9**^**) (M**^**−1**^**)**	***K***_**D **_**ratio**	***K***_**D **_**(×10**^**−9**^**) (M**^**−1**^**)**	***K***_**D **_**ratio**
PAB01 (WT)	12.8	-	1080	-
PAB02 (D36H)	15.5	1.2	1350	1.3
	**pH = 7.4**		**pH = 5.0**	
**Protein**	***K***_**D **_**(×10**^**−5**^**) (M**^**−1**^**)**	***K***_**D **_**ratio**	***K***_**D **_**(×10**^**−5**^**) (M**^**−1**^**)**	***K***_**D **_**ratio**
PAB01 (WT)	2.9	-	ND	-
PAB02 (D36H)	6.7	2.3	ND	-

The binding assay was performed under neutral (pH = 7.4) and acidic (pH = 5.0) conditions using SPR. IgG-Fc or IgG-Fab was immobilized on the surface of a CM5 sensor chip (GE Healthcare).

*K*_D_, equilibrium dissociation constant: *K*_D_ ratio, (*K*_D_ of histidine-substituted PAB variant)/(*K*_D_ of Wild-type PAB or PAZ): ND, not determined.

Next, we analyzed the effects of D36H using the structure of SpA D-domain complexed with IgG-Fab [30]. D36 is conserved in all the SpA domains (A, B, C, D and E). The crystal structure indicates that six residues in IgG-Fab are within 4 Å of D36 in PAB. Therefore, the steric hindrance due to the histidine-mutation of D36 would affect the interaction with IgG-Fab under neutral conditions. Because a positively charged residue (Arg2519) in IgG-Fab is present near D36 in PAB (<3.0 Å), electrostatic repulsion under acidic conditions should be stronger than under neutral conditions.

We also focused on Arg2519 in IgG-Fab as it would be involved in the effects of histidine-mutation of D36. Q32 of PAB is located within 4 Å of Arg2519 of IgG-Fab. The PAB Q32H variant (PAB10) was produced, and the pH value of the IgG elution peaks from the affinity column with PAB10 was found to be significantly higher than those values for PAB01 (WT), PAB07 (Q9H) and PAB08 (Q10H) columns. This suggested that the introduction of histidine-mutations into the IgG-Fab binding interface of PAB was as effective as introducing them into the IgG-Fc binding interface. In addition, the majority of amino acid residues corresponding to Arg2519 of the top 10 therapeutic antibodies are positively charged ones (Additional file 9: Table S4). Therefore, the PAB affinity ligands with D36H mutation should be effective for therapeutic antibody purification.

Although the binding affinity of IgG-Fab for PAB01 (WT) was more than 1000-fold weaker than that of IgG-Fc (Table 4), a possible reason for the remarkable effects of the D36H mutation would be a change in avidity. IgG has four SpA binding sites: two are strong binding sites in the IgG-Fc region and two are weak binding sites in the IgG-Fab region. On an affinity column, since a plurality of interactions for these four sites would bind the IgG molecule, the cooperative effect of interactions between SpA and IgG would be strong. Since the D36H mutation would result in just 2 binding sites instead of 4 in the interaction with IgG, the effect of avidity would likely decrease significantly, thus promoting dissociation under moderately acidic conditions.

### Elution profile of bound IgG from 4 × PAZ variant columns

**Figure 2:**
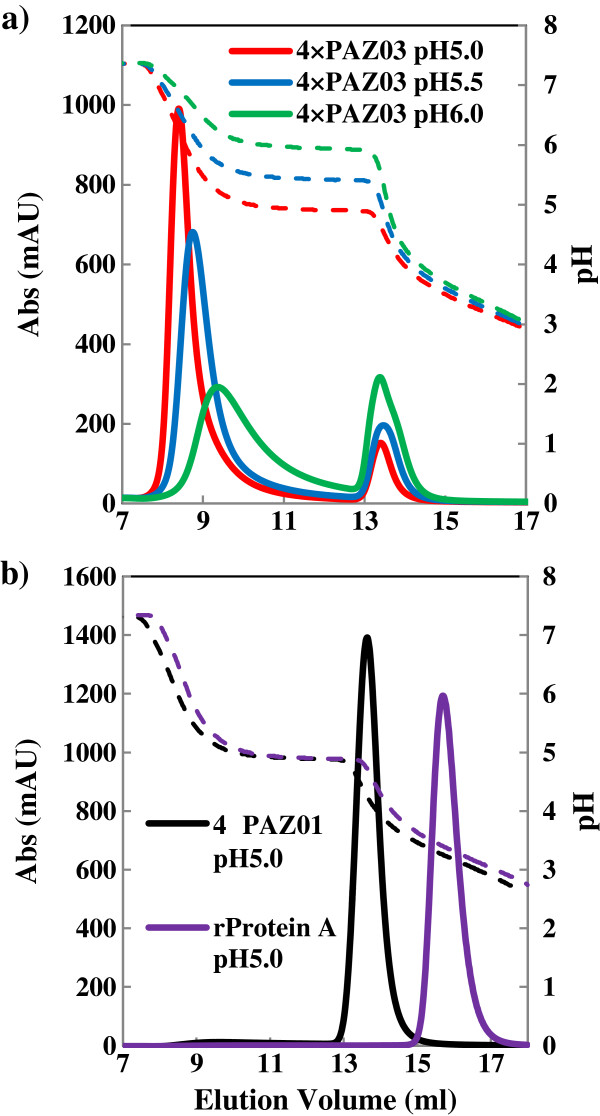
**Elution profile of IgG on affinity columns with immobilized 4 × PAZ variants. (a)** Elution profile of IgG from affinity columns prepared with immobilized 4 × PAZ03. IgG was eluted with pH 5.0, pH 5.5 or pH 6.0 buffer, **(b)** Elution profile of IgG from affinity columns prepared with immobilized 4 × PAZ01 or rProteinA (GE Healthcare). Elution was performed using pH 5.0 buffer. Affinity columns were prepared using the 4 × PAZ variants. The captured IgG on the column was eluted using a buffer with the pH indicated in the Figure. Afterwards, the residual IgG was eluted with a decreasing pH gradient. The ordinate on the right indicates the pH of the solution. The ordinate on the left indicates the absorbance at 280 nm (mAU: milliabsorbance units). The abscissa indicates the elution volume. The solid and dashed lines show the elution patterns of IgG on 4 × PAZ variants or rProteinA (GE Healthcare) columns, as shown in the Figure, and the pH value of the elution buffer, respectively.

An affinity column of tandem SpA (4 × PAZ) consisting of four SpA Z-domains was fabricated using pH-sensitive double substituted PAB variant (PAB03) and its performance was compared with commercially available affinity columns (Figure 2).

IgG was eluted with a stepwise pH decrease. The relative amounts of IgG eluted under mild acidic conditions (pH = 5 ~ 6) was 67% at pH = 6.0, 82% at pH = 5.5 and 90% at pH = 5.0. In contrast, IgG bound to the 4 × PAZ01 column or to a commercially available SpA column (rProtein A) (GE Healthcare) did not elute at pH = 5.0. Although a part of IgG injected onto the 4 × PAZ03 column seemed to flow through, more than the case of the 4 × PAZ01 column, with pH 7.5 binding buffer, the amounts of IgG leaking out were very little, if any, with pH 9.0 binding buffer (Additional file 10: Figure S7). Thus, the 4 × PAZ03 (Q9H, D36H) column would allow the retention of IgG by optimizing the affinity column condition and the elution from the column under much milder pH conditions than previously reported [7,8,11].

## Conclusion

We have shown that a histidine-scanning library combined with a structure-based design was effective in engineering novel pH-sensitive proteins. The positions at which histidine residues occurred with high frequency should be suitable sites for introducing histidine-mutations. Large effects of PAB mutations such as Q9H and Q10H, combined with D36H, were observed. The chromatograms demonstrated that antibodies could be eluted at a higher pH than ever reported previously when the protein A variants developed in this study were used as affinity ligands. The interactions between SpA and IgG-Fab are apparently important for the behavior of IgG bound on a SpA affinity column, and alterations in the affinity of the ligands for IgG-Fab affected the elution condition for bound IgG. Therefore, not only the interactions with IgG-Fc but also with IgG-Fab should be considered when designing a new affinity ligand for IgG purification.

## Materials and methods

### Histidine-scanning library design

Distances from positively charged residues (histidine, lysine and arginine residues) on IgG1-Fc to all residues of PAB in the crystal structure of the complex of PAB bound to IgG1-Fc [pdb: 1FC2] (Table 1) [20] were calculated using the program CONTACT in CCP4 suite [31]. The solvent-accessible surface area (SASA) was calculated for the subunit structure of PAB in the complex. The extent of solvent exposure of all residues of PAB was determined by the equation (Table 1) fSASA (a fractional solvent-accessible surface area) = SASA (native)/SASA (denatured), where a Gly-Xaa-Gly tripeptide model was assumed to adopt the denatured structure. The calculation was performed using surface Racer 3.0 [32].

Gene fragments for constructing a mutated PAB library were prepared with chemically synthesized oligonucleotides (Additional file 11: Table S1), purchased from Rikaken, by the overlap extension method using combinations of these oligonucleotides. At the mutation position of the oligonucleotide, we introduced mixed codons comprising three mixed nucleotides, encoding either the wild-type residue or a histidine residue. However, genes encoding neither the wild-type nor a histidine residue at that position were also produced, since the codon was a random mixture of three nucleotides. Consequently, genes encoding three or four different kinds of residues were obtained for several mutation positions.

The gene fragments thus prepared were treated with restriction enzymes *Eco*R I and *Hind* III and linked to the 3′-terminal side of the g10 gene on the T7 phage genome through ligation reaction (16°C, 16 hr) with T7 phage vector (T7Select1-1b) (Novagen). The linked T7 phage genome was subjected to an *in vitro* T7 phage particle packaging reaction (22°C, 2 hr) to prepare the initial library.

An *E. coli* BLT5403 strain cultured in 200 mL LB medium to O.D.600 = 1.0 was infected with this initial library, and a T7 phage library was collected by amplification. About 4 hours after infection, the amplified phages were recovered (T7 phages have bacteriolytic ability and are thus released from the bacterial body) in the supernatant after centrifugation. To the supernatant, 1/6 volume of 50% polyethylene glycol (PEG, molecular weight: 8000) and 1/10 volume of 5 M NaCl were added, and the mixture was stirred at 4°C overnight. The PEG-precipitated phages were partially purified by centrifugation. Then, the phages were lysed by repeated pipetting in TBST buffer (10 mM Tris–HCl (pH = 7.5), 150 mM NaCl, and 0.1% Tween 20) and the resultant suspension was filtered through a 0.22 μm filter to obtain the phage library displaying the mutated PAB. This is referred to as the initial phage library.

### Phage panning and selection

Human monoclonal IgG1 antibody (Chugai) was biotinylated using NHS-biotin (Roche) according to the manufacturer’s protocol. The biotinylated monoclonal antibody was mixed with streptavidin magnetic beads (Promega) to immobilize the antibodies on the magnetic beads.

For the binding step, the suspension of the phage library (10^9^ ~ 10^11^ phage/mL titer) displaying the mutated PAB prepared above was added to the biotinylated antibody-immobilized magnetic beads, and the mixture was shaken at 25°C for 1 hour. Afterwards, the beads with bound phages were pulled down by using a magnetic stand (Promega). The beads were washed 10 to 20 times with TBST buffer to remove antibody-unbound phages.

For the elution step, phages specifically bound with the antibodies were recovered by adding a 50 mM sodium acetate solution (pH = 5.0). The eluted phages were subjected to the infection/amplification/PEG-precipitation operation described in the preceding paragraph to prepare the phage library displaying the mutated PAB. This is referred to as the phage library after the first round.

The operation described above, i.e., the screening experiment, was repeated 5 times in total. The eluting solution used in the screening experiment from the second round onwards was 50 mM sodium acetate (pH = 6.0). The selective pressure of screening was changed at each stage by gradually decreasing the amount of biotinylated antibody-immobilized magnetic beads.

### Protein preparation

Expression vectors for single or double histidine-substituted PAB variants were prepared using a QuickChange™ site-directed mutagenesis kit (Stratagene). The expression vector for wild-type PAB (pET16-b) was used as the template. The primer sequences (Rikaken) used for this procedure are shown in Additional file 11: Table S1.

The expression vectors for the tandem SpA Z-domain (4 × PAZ) variants comprised artificial pET16-b vectors containing four sets of the SpA Z-domain gene and the C-terminal cysteine codon and were purchased from Genscript.

*E. coli* strain BL21 (DE3) was transformed with these plasmid vectors and cultured in 2 × YT medium containing 100 μg/ml ampicillin to O.D.600 = 0.8 to 1.0 at 37°C. Recombinant gene expression of PAB and the 4 × PAZ variants was induced by the addition of isopropylthio-β-D-galactopyranoside (IPTG) to a final concentration of 1 mM, then the transformants were further cultured at 37°C for 2 hours. The bacteria harvested were suspended in TBST buffer and homogenized by ultrasonication. PAB and 4 × PAZ variants were purified from the cell lysate by IgG Sepharose 6 Fast Flow chromatography (GE Healthcare). The purity of the sample was checked with Tricine-SDS-PAGE.

### Affinity chromatography

To prepare a PAB variant-immobilized affinity column, a purified PAB variant was applied to a HiTrap NHS HP column (GE Healthcare), incubated at room temperature (overnight), and then washed with 0.5 M acetic acid (pH 2.5). Affinity chromatography was performed with an AKTA purifier (GE Healthcare) using the columns as above. Human IgG1 was injected onto the column equilibrated with TBST buffer. The captured IgG was eluted with a decreasing pH gradient by using 50 mM sodium citrate (pH = 7.0) and 0.5 M acetic acid (pH = 2.5) (Table 2, Additional file 5: Figure S4). The chromatograms obtained were processed using UNICORN version 4.12 (GE Healthcare).To prepare a tandem SpA Z-domain (4 × PAZ) variant-immobilized affinity column, a purified 4 × PAZ variant was chemically coupled to sepharose resin through the sulfhydryl group of the C-terminal cysteine using a SulfoLink Immobilization kit (Pierce). Affinity chromatography was performed as above. The captured IgG was eluted using 50 mM sodium citrate (pH = 5.0, 5.5 or 6.0). Afterwards, the residual IgG on the 4 × PAZ column was eluted with a decreasing pH gradient by using 50 mM sodium citrate (pH = 5.0, 5.5, or 6.0) and 0.5 M acetic acid (pH = 2.5) (Figure 2).

### Stability analysis

PAB variants were dissolved in 50 mM sodium phosphate (pH = 7.0). Circular dichroism (CD) spectra were obtained on a J-805 spectropolarimeter (JASCO) at various temperatures. CD melting curves were obtained by monitoring the ellipticity at 222 nm with increasing temperature from 278 to 373 K at a heating rate of 1.0 K/min. The thermodynamic parameters of proteins for equilibrium unfolding were obtained through a fitting calculation using a two-state transition model [33] on the assumption that heat capacity changes (Δ*C*_*p*_) of all proteins are constant and fixed to the value of the wild-type domain (Δ*C*_*p*_ = 2.643 kJ/mol•K) (Table 2). The value of Δ*C*_*p*_ was estimated using the linear relationship between molecular weight and Δ*C*_*p*_ displayed by various proteins [34]. Numerical fitting calculations were carried out using IGOR software (Wavemetrics). Other procedures were the same as described previously [35].

### Binding affinity analysis

Binding analyses of a PAB variant to IgG, IgG-Fc and IgG-Fab were performed using a Biacore T100 (GE Healthcare). Either 4000 resonance units (RU) of human monoclonal IgG1, 1250 RU of the Fab region of human polyclonal antibody (Jackson Immunoresearch), or 1250 RU of the Fc region of human polyclonal antibody (Jackson Immunoresearch) was immobilized on the surface of a CM5 sensor chip (GE Healthcare) using amine coupling chemistry. A binding assay was performed under neutral and acidic conditions as follows: (1) 10 mM HEPES (pH = 7.4), 150 mM NaCl and Tween 20 (0.05%); (2) 10 mM sodium acetate (pH = 5.0), 150 mM NaCl and Tween 20 (0.05%). The binding data was fitted to a 1:1 binding model to determine the dissociation constant (*K*_D_) using Biacore T100 Evaluation software (Tables 3 and 4).

## Endnote

*In this study, ligand proteins with favorable pH sensitivities are referred to as “pH-sensitive”. **∆pH = (the pH value at which IgG eluted from mutant column) - (the pH value at which IgG eluted from wild-type column).

## Abbreviations

SpA: *Staphylococcal* protein A; Ig: Immunoglobulin; PAB: *Staphylococcal* protein A B-domain; PAZ: *Staphylococcal* protein A Z-domain.

## Competing interests

We declare that all authors are inventors on a pending patent using these *Staphylococcal* protein A variants.

## Authors’ contributions

MT conducted experiments, analyzed data, and co-wrote the manuscript. HW and AO conducted experiments and analyzed data. SH designed the study, analyzed data and co-wrote the manuscript. All authors read and approved the final manuscript.

## Supplementary Material

Additional file 1: Table S2 Eighteen unique sequences of PAB variants after the final round. Amino acid residues at the indicated position number are shown for PAB_wild-type_ and for 18 unique sequences from the final round, PAB_T7phage_01-18. Click here for file

Additional file 2: Figure S1 Frequency of occurrence of amino acid residues for each mutation position (5F, 6N, 9Q, 10Q, 11N and 13F). The frequency of occurrence of amino acid residues was calculated from the determined sequences of PAB variants after each round. The title of each section of the Figure, such as Position 5F, indicates the wild-type residue (in this case, F) of the mutation position. The ordinate indicates the percentage of the frequency of occurrence. The abscissa indicates the round number. Wild-type residue (blue), Histidine residue (red), Non-wild-type and non–histidine residues (green or purple). Click here for file

Additional file 3: Figure S2 Frequency of occurrence of amino acid residues for each mutation position (14Y, 15E, 17L, 24E, 25E and 27R). see the caption in Additional file 2: Figure S1. Click here for file

Additional file 4: Figure S3 Frequency of occurrence of amino acid residues for each mutation position (28N, 31I, Q32, 35K and 36D). see the caption in Additional file 2: Figure S1. Click here for file

Additional file 5: Figure S4 Elution profiles of IgG on affinity columns with immobilized PAB variants. Affinity columns were prepared using the PAB variants. The captured IgG on the column was eluted with a decreasing pH gradient. The ordinate on the right indicates the pH of the solution. The ordinate on the left indicates the absorbance at 280 nm (mAU: milliabsorbance units). The abscissa indicates the elution volume. The solid and dashed lines show the elution patterns of IgG on affinity columns with immobilized PAB variants as shown in the Figure and the pH value of the elution buffer, respectively. Click here for file

Additional file 6: Table S3 PAB variants data by mutational experiments [7, 11, 21-24] and by molecular simulation calculations [25-26]. The binding affinity of IgG for PAB variants was determined using ELISA and SPR. None: Almost the same binding affinity, Very small: 2-fold to < 5-fold decrease, Small: 5-fold to < 10-fold decrease, Large: 10-fold to < 100-fold decrease, Very large: 100-fold decrease <. Click here for file

Additional file 7: Figure S5 Circular dichroism melting curves of PAB variants. (a) The curves of the single histidine substituted PAB variants. (b) The curves of the double histidine substituted PAB variants. Circular dichroism melting curves were obtained by monitoring the ellipticity at 222 nm with increasing temperature on a J-805 spectropolarimeter (JASCO). The mole fractions of the proteins in an unfolded state (thick lines) are shown as a function of temperature. Theoretical curves (thin lines) were calculated using a two-state equilibrium transition model. Click here for file

Additional file 8: Figure S6 Binding efficiency of PAB variants. The PAB variant (about 72 μg) was immobilized on NHS-activated agarose gel. The IgG (about 1mg) solution was added to the PAB variant immobilized agarose gel, and the mixture was shaken at 25°C for 30 min in pH 9.0 buffer (25 mM Tris–HCl (pH=9.0), 2.5 M NaCl, and 0.1% Tween 20). The amounts of IgG in supernatant after centrifugation were determined and the binding efficiency was calculated. The ordinate indicates the binding efficiency. Click here for file

Additional file 9: Table S4 Amino acid residues of therapeutic antibodies related to the effects of D36H. In this study, Arg2519 of IgG-Fab was found to be related to the effects of D36H mutation. Of top 10 therapeutic antibodies in 2010, 80% of the amino acid residues corresponding to Arg2519 are positively charged residues. Sales rank of therapeutic antibodies in 2010 are cited from the article (John G. Elvin *et al.* International Journal of Pharmaceutics 440 (2013) 83– 98). Click here for file

Additional file 10: Figure S7 Elution profile of IgG on affinity columns with immobilized 4×PAZ variants. (a) Elution profile of IgG from affinity columns prepared with immobilized 4×PAZ01 or 4×PAZ03. IgG was bound with pH 7.5 buffer (25 mM Tris–HCl (pH=7.5), 150 mM NaCl, and 0.1% Tween 20) at a flow rate of 0.5 mL/min, (b) Elution profile of IgG from affinity columns prepared with immobilized 4×PAZ01 or 4×PAZ03. IgG was bound with pH 7.5 buffer (25 mM Tris–HCl (pH=7.5), 150 mM NaCl, and 0.1% Tween 20) at a flow rate of 0.1 mL/min, (c) Elution profile of IgG from affinity columns prepared with immobilized 4×PAZ01 or 4×PAZ03. IgG was bound with pH 9.0 buffer (25 mM Tris–HCl (pH=9.0), 2.5 M NaCl, and 0.1% Tween 20) at a flow rate of 0.1 mL/min. Affinity columns were prepared using the 4×PAZ variants. The captured IgG on the column was eluted using a buffer with the pH indicated in the Figure. Afterwards, the residual IgG was eluted with a decreasing pH gradient. The ordinate on the right indicates the pH of the solution. The ordinate on the left indicates the absorbance at 280 nm (mAU: milliabsorbance units). The abscissa indicates the elution volume. The solid and dashed lines show the elution patterns of IgG on 4×PAZ variant columns, as shown in the Figure, and the pH value of the elution buffer, respectively. The inlets show the elution patterns with magnified scale from 0 to 8 mL. Click here for file

Additional file 11: Table S1 List of primers. Primers 1~4 indicate the oligonucleotides for constructing a histidine-scanning library. Primers 5~12 indicate the oligonucleotides for introducing histidine-mutations. The following abbreviations are used for mixed bases: R=(A or G), Y=(C or T), M=(A or C), K=(G or T), S=(G or C) and W=(A or T). Click here for file
