# Supplementary material for: Engineered protein A ligands, derived from a histidine-scanning library, facilitate the affinity purification of IgG under mild acidic conditions
Source: J Biol Eng. 2014 Jul 1;8:15. doi: 10.1186/1754-1611-8-15 (PMC4107488; doi:10.1186/1754-1611-8-15)
Supplement: Additional file 1: Table S2 — Eighteen unique sequences of PAB variants after the final round. Amino acid residues at the indicated position number are shown for PABwild-type and for 18 unique sequences from the final round, PABT7phage01-18. [file 1754-1611-8-15-S1.docx]

**Table S2. Eighteen unique sequences of PAB variants after the final round.**

Amino acid residues at the indicated position number are shown for PAB_wild-type_ and for 18 unique sequences from the final round, PAB_T7phage_01-18.

| **Name** | **Position Number** | | | | | | | | | | | | | | | | | **sequenced  clones** |
| --- | --- | --- | --- | --- | --- | --- | --- | --- | --- | --- | --- | --- | --- | --- | --- | --- | --- | --- |
|  | **5** | **6** | **9** | **10** | **11** | **13** | **14** | **15** | **17** | **24** | **25** | **27** | **28** | **31** | **32** | **35** | **36** |  |
| **PAB _Wild-type_** | **F** | **N** | **Q** | **Q** | **N** | **F** | **Y** | **E** | **L** | **E** | **E** | **R** | **N** | **I** | **Q** | **K** | **D** | **-** |
| **PAB_T7Phage_01** | **H** | **N** | **H** | **H** | **H** | **L** | **Y** | **H** | **L** | **E** | **D** | **H** | **H** | **I** | **Q** | **H** | **H** | **61** |
| **PAB_T7Phage_02** | **F** | **H** | **Q** | **Q** | **H** | **F** | **Y** | **D** | **L** | **Q** | **H** | **R** | **N** | **I** | **Q** | **K** | **D** | **10** |
| **PAB_T7Phage_03** | **H** | **N** | **H** | **H** | **H** | **F** | **Y** | **H** | **L** | **E** | **H** | **H** | **H** | **L** | **Q** | **K** | **D** | **7** |
| **PAB_T7Phage_04** | **L** | **N** | **Q** | **Q** | **N** | **Y** | **Y** | **E** | **Q** | **E** | **D** | **R** | **N** | **N** | **Q** | **H** | **H** | **1** |
| **PAB_T7Phage_05** | **H** | **N** | **H** | **H** | **N** | **F** | **Y** | **Q** | **L** | **Q** | **E** | **H** | **H** | **I** | **H** | **H** | **H** | **1** |
| **PAB_T7Phage_06** | **H** | **N** | **H** | **H** | **N** | **Y** | **Y** | **D** | **Q** | **E** | **D** | **H** | **H** | **H** | **H** | **H** | **H** | **1** |
| **PAB_T7Phage_07** | **L** | **H** | **H** | **Q** | **N** | **H** | **H** | **E** | **L** | **H** | **H** | **H** | **N** | **I** | **H** | **H** | **H** | **1** |
| **PAB_T7Phage_08** | **F** | **N** | **H** | **H** | **N** | **F** | **Y** | **Q** | **L** | **E** | **E** | **H** | **N** | **I** | **Q** | **H** | **H** | **1** |
| **PAB_T7Phage_09** | **H** | **N** | **H** | **H** | **N** | **L** | **Y** | **H** | **L** | **E** | **H** | **H** | **N** | **L** | **Q** | **H** | **H** | **1** |
| **PAB_T7Phage_10** | **F** | **H** | **Q** | **Q** | **N** | **F** | **Y** | **E** | **L** | **H** | **Q** | **R** | **N** | **I** | **H** | **K** | **H** | **1** |
| **PAB_T7Phage_11** | **L** | **N** | **H** | **Q** | **N** | **H** | **Y** | **D** | **L** | **D** | **D** | **H** | **N** | **N** | **H** | **H** | **H** | **1** |
| **PAB_T7Phage_12** | **H** | **N** | **H** | **H** | **H** | **L** | **Y** | **H** | **L** | **Q** | **D** | **H** | **H** | **L** | **Q** | **H** | **H** | **1** |
| **PAB_T7Phage_13** | **H** | **N** | **H** | **H** | **H** | **L** | **Y** | **H** | **L** | **E** | **Q** | **H** | **H** | **L** | **Q** | **H** | **H** | **1** |
| **PAB_T7Phage_14** | **L** | **N** | **Q** | **H** | **H** | **F** | **H** | **Q** | **H** | **H** | **Q** | **R** | **N** | **H** | **H** | **H** | **H** | **1** |
| **PAB_T7Phage_15** | **H** | **N** | **H** | **H** | **H** | **L** | **Y** | **Q** | **L** | **E** | **E** | **H** | **H** | **L** | **Q** | **H** | **H** | **1** |
| **PAB_T7Phage_16** | **H** | **H** | **H** | **H** | **H** | **L** | **Y** | **H** | **L** | **E** | **D** | **H** | **H** | **I** | **Q** | **H** | **H** | **1** |
| **PAB_T7Phage_17** | **Y** | **H** | **Q** | **H** | **N** | **L** | **Y** | **D** | **L** | **Q** | **D** | **R** | **H** | **L** | **H** | **H** | **H** | **1** |
| **PAB_T7Phage_18** | **H** | **N** | **H** | **H** | **H** | **L** | **Y** | **H** | **L** | **E** | **E** | **H** | **H** | **L** | **Q** | **H** | **H** | **1** |
