# Supplementary material for: Engineered protein A ligands, derived from a histidine-scanning library, facilitate the affinity purification of IgG under mild acidic conditions
Source: J Biol Eng. 2014 Jul 1;8:15. doi: 10.1186/1754-1611-8-15 (PMC4107488; doi:10.1186/1754-1611-8-15)
Supplement: Additional file 2: Figure S1 — Frequency of occurrence of amino acid residues for each mutation position (5F, 6N, 9Q, 10Q, 11N and 13F). The frequency of occurrence of amino acid residues was calculated from the determined sequences of PAB variants after each round. The title of each section of the Figure, such as Position 5F, indicates the wild-type residue (in this case, F) of the mutation position. The ordinate indicates the percentage of the frequency of occurrence. The abscissa indicates the round number. Wild-type residue (blue), Histidine residue (red), Non-wild-type and non–histidine residues (green or purple). [file 1754-1611-8-15-S2.pptx]

## Slide 1
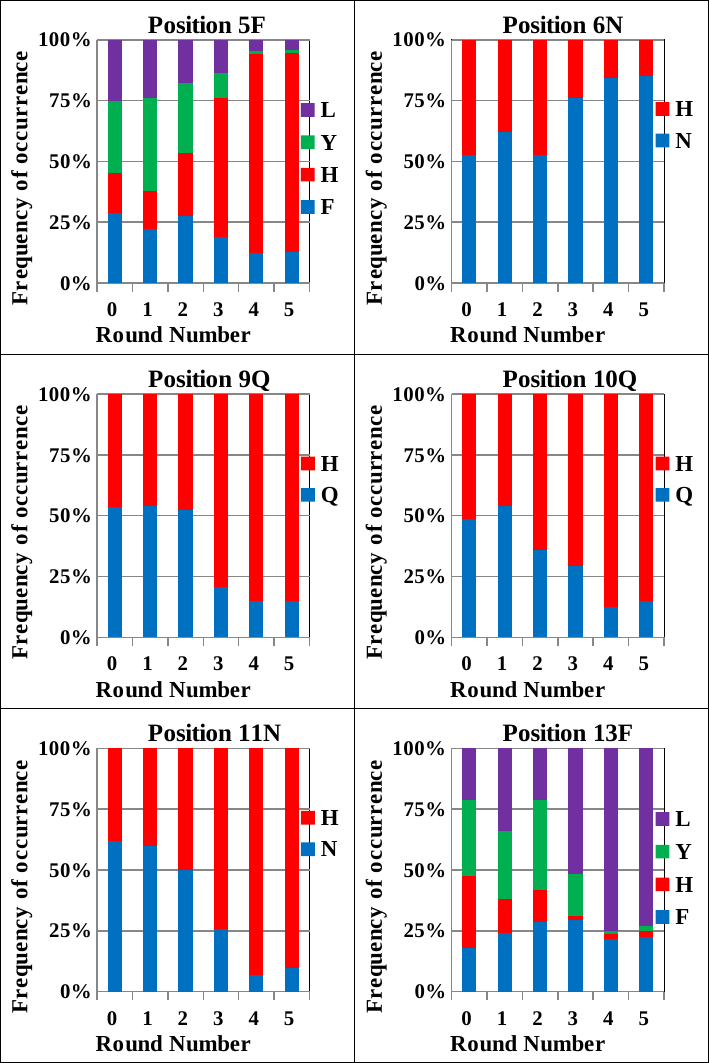

### Chart: Position 5F
| Category | F | H | Y | L |
|---|---|---|---|---|
| 0 | 28.57142857142857 | 16.666666666666664 | 29.761904761904788 | 25.0 |
| 1 | 22.0 | 16.0 | 38.0 | 24.0 |
| 2 | 27.380952380952383 | 26.190476190476193 | 28.57142857142857 | 17.857142857142833 |
| 3 | 18.965517241379235 | 56.896551724137986 | 10.344827586206897 | 13.793103448275843 |
| 4 | 12.5 | 81.81818181818161 | 1.1363636363636365 | 4.545454545454546 |
| 5 | 12.903225806451612 | 81.72043010752665 | 1.0752688172042995 | 4.301075268817197 |
### Chart: Position 6N
| Category | N | H |
|---|---|---|
| 0 | 52.38095238095239 | 47.61904761904751 |
| 1 | 62.0 | 38.0 |
| 2 | 52.38095238095239 | 47.61904761904751 |
| 3 | 75.86206896551727 | 24.137931034482797 |
| 4 | 84.0909090909091 | 15.90909090909092 |
| 5 | 84.94623655913979 | 15.053763440860216 |
### Chart: Position 9Q
| Category | Q | H |
|---|---|---|
| 0 | 53.57142857142851 | 46.42857142857149 |
| 1 | 54.0 | 46.0 |
| 2 | 52.38095238095239 | 47.61904761904751 |
| 3 | 20.689655172413794 | 79.31034482758604 |
| 4 | 14.772727272727286 | 85.22727272727273 |
| 5 | 15.053763440860216 | 84.94623655913979 |
### Chart: Position 10Q
| Category | Q | H |
|---|---|---|
| 0 | 48.80952380952381 | 51.19047619047621 |
| 1 | 54.0 | 46.0 |
| 2 | 35.714285714285715 | 64.28571428571429 |
| 3 | 29.310344827586203 | 70.68965517241361 |
| 4 | 12.5 | 87.5 |
| 5 | 15.053763440860216 | 84.94623655913979 |
### Chart: Position 11N
| Category | N | H |
|---|---|---|
| 0 | 61.904761904761905 | 38.0952380952381 |
| 1 | 60.0 | 40.0 |
| 2 | 50.0 | 50.0 |
| 3 | 25.862068965517242 | 74.13793103448258 |
| 4 | 6.818181818181809 | 93.18181818181817 |
| 5 | 9.67741935483872 | 90.32258064516128 |
### Chart: Position 13F
| Category | F | H | Y | L |
|---|---|---|---|---|
| 0 | 17.857142857142833 | 29.761904761904788 | 30.952380952380917 | 21.428571428571427 |
| 1 | 24.0 | 14.000000000000002 | 28.000000000000004 | 34.0 |
| 2 | 28.57142857142857 | 13.0952380952381 | 36.904761904761905 | 21.428571428571427 |
| 3 | 29.310344827586203 | 1.7241379310344827 | 17.24137931034483 | 51.72413793103451 |
| 4 | 21.590909090909086 | 2.2727272727272787 | 1.1363636363636365 | 75.0 |
| 5 | 22.58064516129032 | 2.1505376344086025 | 2.1505376344086025 | 73.1182795698925 |
