# Supplementary material for: Engineered protein A ligands, derived from a histidine-scanning library, facilitate the affinity purification of IgG under mild acidic conditions
Source: J Biol Eng. 2014 Jul 1;8:15. doi: 10.1186/1754-1611-8-15 (PMC4107488; doi:10.1186/1754-1611-8-15)
Supplement: Additional file 3: Figure S2 — Frequency of occurrence of amino acid residues for each mutation position (14Y, 15E, 17L, 24E, 25E and 27R). see the caption in Additional file 2: Figure S1. [file 1754-1611-8-15-S3.pptx]

## Slide 1
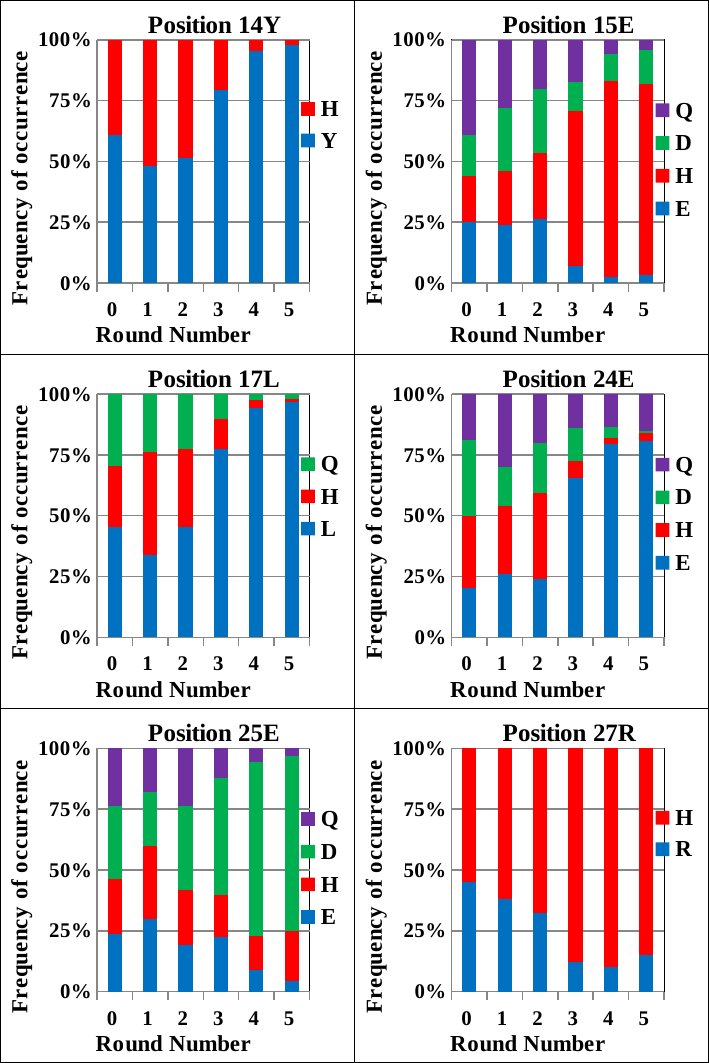

### Chart: Position 14Y
| Category | Y | H |
|---|---|---|
| 0 | 60.71428571428571 | 39.285714285714285 |
| 1 | 48.0 | 52.0 |
| 2 | 51.19047619047621 | 48.80952380952381 |
| 3 | 79.31034482758598 | 20.689655172413794 |
| 4 | 95.45454545454547 | 4.545454545454546 |
| 5 | 97.84946236559138 | 2.1505376344086025 |
### Chart: Position 15E
| Category | E | H | D | Q |
|---|---|---|---|---|
| 0 | 25.0 | 19.047619047619026 | 16.666666666666664 | 39.285714285714285 |
| 1 | 24.0 | 22.0 | 26.0 | 28.000000000000004 |
| 2 | 26.190476190476193 | 27.380952380952383 | 26.190476190476193 | 20.238095238095227 |
| 3 | 6.896551724137931 | 63.79310344827599 | 12.068965517241379 | 17.24137931034483 |
| 4 | 2.27272727272728 | 80.68181818181817 | 11.363636363636388 | 5.681818181818183 |
| 5 | 3.2258064516129052 | 78.4946236559143 | 13.978494623655926 | 4.301075268817195 |
### Chart: Position 17L
| Category | L | H | Q |
|---|---|---|---|
| 0 | 45.238095238095354 | 25.0 | 29.76190476190479 |
| 1 | 34.0 | 42.0 | 24.0 |
| 2 | 45.238095238095354 | 32.14285714285715 | 22.61904761904763 |
| 3 | 77.58620689655149 | 12.068965517241379 | 10.344827586206897 |
| 4 | 94.31818181818156 | 3.4090909090909087 | 2.27272727272728 |
| 5 | 96.77419354838725 | 1.075268817204299 | 2.1505376344086025 |
### Chart: Position 24E
| Category | E | H | D | Q |
|---|---|---|---|---|
| 0 | 20.238095238095227 | 29.76190476190479 | 30.95238095238091 | 19.047619047619026 |
| 1 | 26.0 | 28.000000000000004 | 16.0 | 30.0 |
| 2 | 23.809523809523743 | 35.714285714285715 | 20.238095238095227 | 20.238095238095227 |
| 3 | 65.5172413793105 | 6.896551724137931 | 13.79310344827584 | 13.79310344827584 |
| 4 | 79.54545454545452 | 2.27272727272728 | 4.545454545454546 | 13.636363636363635 |
| 5 | 80.64516129032258 | 3.2258064516129052 | 1.075268817204299 | 15.053763440860216 |
### Chart: Position 25E
| Category | E | H | D | Q |
|---|---|---|---|---|
| 0 | 23.809523809523743 | 22.61904761904763 | 29.76190476190479 | 23.809523809523743 |
| 1 | 30.0 | 30.0 | 22.0 | 18.0 |
| 2 | 19.047619047619026 | 22.61904761904763 | 34.523809523809526 | 23.809523809523743 |
| 3 | 22.413793103448278 | 17.24137931034483 | 48.275862068965516 | 12.068965517241379 |
| 4 | 9.0909090909091 | 13.636363636363635 | 71.5909090909091 | 5.681818181818183 |
| 5 | 4.301075268817195 | 20.43010752688172 | 72.04301075268812 | 3.2258064516129052 |
### Chart: Position 27R
| Category | R | H |
|---|---|---|
| 0 | 45.238095238095354 | 54.761904761904766 |
| 1 | 38.0 | 62.0 |
| 2 | 32.14285714285715 | 67.85714285714282 |
| 3 | 12.068965517241379 | 87.93103448275863 |
| 4 | 10.227272727272698 | 89.77272727272704 |
| 5 | 15.053763440860216 | 84.94623655913979 |
