# Supplementary material for: Engineered protein A ligands, derived from a histidine-scanning library, facilitate the affinity purification of IgG under mild acidic conditions
Source: J Biol Eng. 2014 Jul 1;8:15. doi: 10.1186/1754-1611-8-15 (PMC4107488; doi:10.1186/1754-1611-8-15)
Supplement: Additional file 4: Figure S3 — Frequency of occurrence of amino acid residues for each mutation position (28N, 31I, Q32, 35K and 36D). see the caption in Additional file 2: Figure S1. [file 1754-1611-8-15-S4.pptx]

## Slide 1
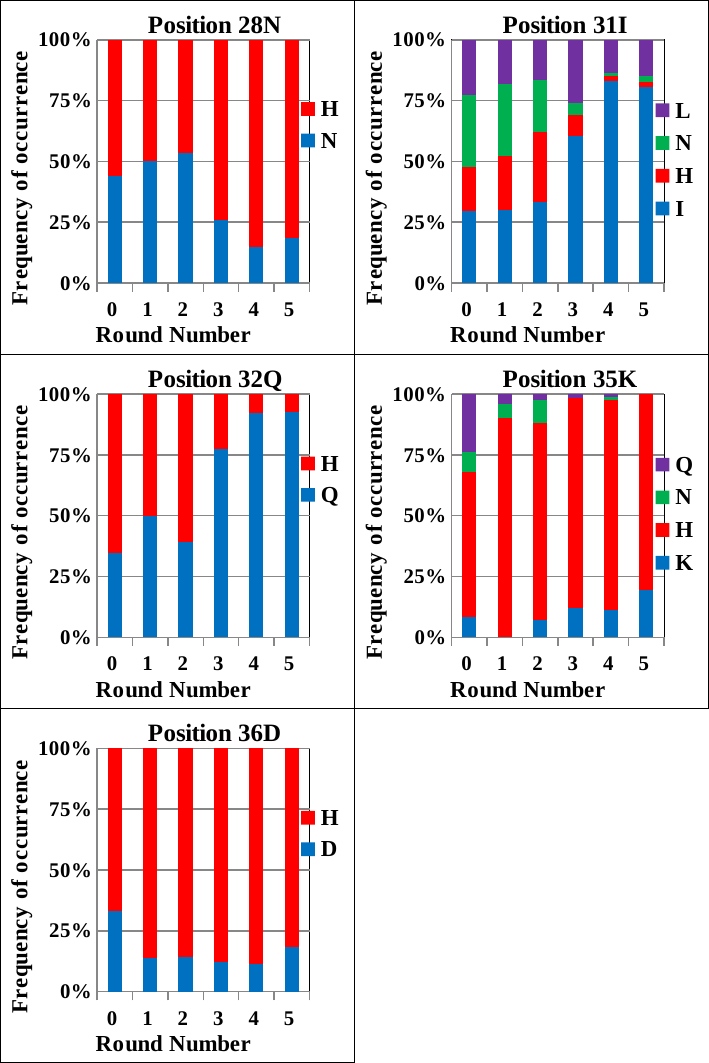

### Chart: Position 28N
| Category | N | H |
|---|---|---|
| 0 | 44.047619047619044 | 55.952380952380956 |
| 1 | 50.0 | 50.0 |
| 2 | 53.57142857142849 | 46.42857142857151 |
| 3 | 25.862068965517242 | 74.13793103448253 |
| 4 | 14.772727272727291 | 85.22727272727273 |
| 5 | 18.27956989247308 | 81.72043010752658 |
### Chart: Position 31I
| Category | I | H | N | L |
|---|---|---|---|---|
| 0 | 29.76190476190479 | 17.85714285714283 | 29.76190476190479 | 22.61904761904763 |
| 1 | 30.0 | 22.0 | 30.0 | 18.0 |
| 2 | 33.33333333333333 | 28.57142857142857 | 21.428571428571427 | 16.666666666666664 |
| 3 | 60.34482758620674 | 8.620689655172415 | 5.172413793103448 | 25.862068965517242 |
| 4 | 82.95454545454547 | 2.27272727272728 | 1.1363636363636365 | 13.636363636363635 |
| 5 | 80.64516129032258 | 2.1505376344086025 | 2.1505376344086025 | 15.053763440860216 |
### Chart: Position 32Q
| Category | Q | H |
|---|---|---|
| 0 | 34.523809523809526 | 65.47619047619048 |
| 1 | 50.0 | 50.0 |
| 2 | 39.285714285714285 | 60.71428571428571 |
| 3 | 77.58620689655149 | 22.413793103448278 |
| 4 | 92.04545454545452 | 7.954545454545445 |
| 5 | 92.47311827956989 | 7.526881720430108 |
### Chart: Position 35K
| Category | K | H | N | Q |
|---|---|---|---|---|
| 0 | 8.333333333333332 | 59.523809523809526 | 8.333333333333332 | 23.809523809523743 |
| 1 | 0.0 | 90.0 | 6.0 | 4.0 |
| 2 | 7.1428571428571415 | 80.95238095238074 | 9.523809523809524 | 2.380952380952381 |
| 3 | 12.068965517241379 | 86.206896551724 | 0.0 | 1.7241379310344827 |
| 4 | 11.363636363636388 | 86.36363636363629 | 1.1363636363636365 | 1.1363636363636365 |
| 5 | 19.354838709677466 | 80.64516129032258 | 0.0 | 0.0 |
### Chart: Position 36D
| Category | D | H |
|---|---|---|
| 0 | 33.33333333333333 | 66.66666666666666 |
| 1 | 14.000000000000002 | 86.0 |
| 2 | 14.285714285714286 | 85.71428571428572 |
| 3 | 12.068965517241379 | 87.93103448275863 |
| 4 | 11.363636363636388 | 88.63636363636364 |
| 5 | 18.27956989247308 | 81.72043010752658 |
