# Supplementary figures and images for: Engineered protein A ligands, derived from a histidine-scanning library, facilitate the affinity purification of IgG under mild acidic conditions
Source: J Biol Eng. 2014 Jul 1;8:15. doi: 10.1186/1754-1611-8-15 (PMC4107488; doi:10.1186/1754-1611-8-15)

## Slide 1
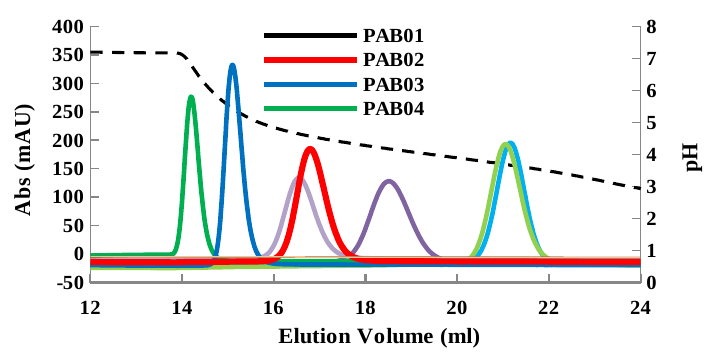

### Chart
| Category | | | | | | | | | |
|---|---|---|---|---|---|---|---|---|---|

Supplement: Additional file 5: Figure S4 — Elution profiles of IgG on affinity columns with immobilized PAB variants. Affinity columns were prepared using the PAB variants. The captured IgG on the column was eluted with a decreasing pH gradient. The ordinate on the right indicates the pH of the solution. The ordinate on the left indicates the absorbance at 280 nm (mAU: milliabsorbance units). The abscissa indicates the elution volume. The solid and dashed lines show the elution patterns of IgG on affinity columns with immobilized PAB variants as shown in the Figure and the pH value of the elution buffer, respectively. [file 1754-1611-8-15-S5.pptx]

## Slide 1
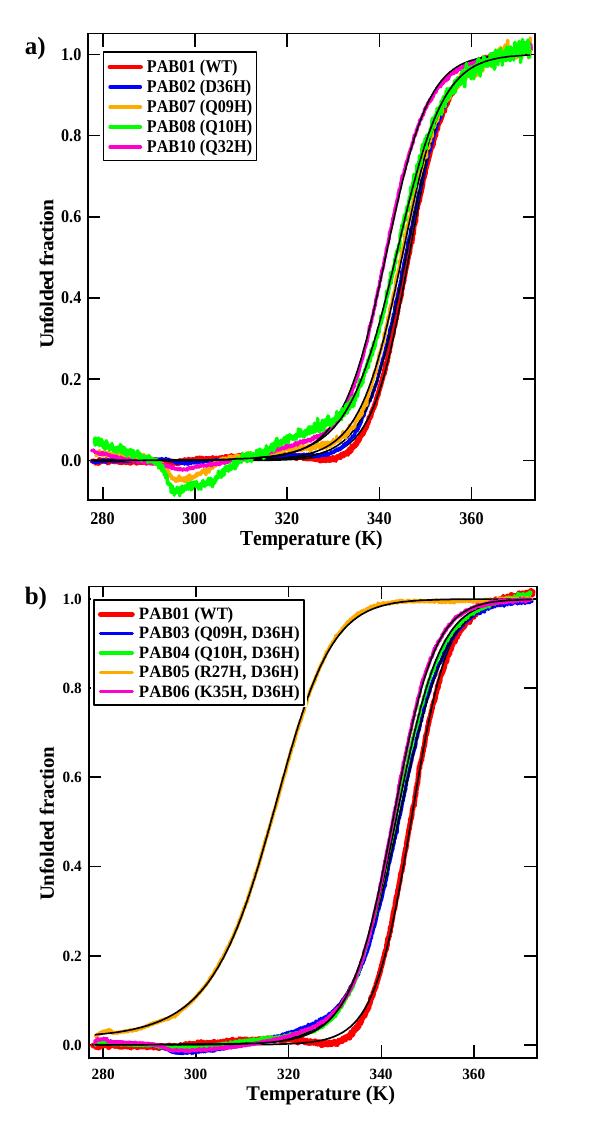

a)
b)

Supplement: Additional file 7: Figure S5 — Circular dichroism melting curves of PAB variants. (a) The curves of the single histidine substituted PAB variants. (b) The curves of the double histidine substituted PAB variants. Circular dichroism melting curves were obtained by monitoring the ellipticity at 222 nm with increasing temperature on a J-805 spectropolarimeter (JASCO). The mole fractions of the proteins in an unfolded state (thick lines) are shown as a function of temperature. Theoretical curves (thin lines) were calculated using a two-state equilibrium transition model. [file 1754-1611-8-15-S7.pptx]

## Slide 1
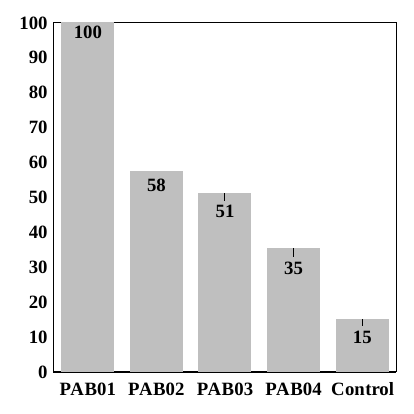

### Chart
| Category | |
|---|---|
| PAB01 | 100.0 |
| PAB02 | 57.53846153846155 |
| PAB03 | 51.07692307692309 |
| PAB04 | 35.38461538461539 |
| Control | 15.076923076923093 |

Supplement: Additional file 8: Figure S6 — Binding efficiency of PAB variants. The PAB variant (about 72 μg) was immobilized on NHS-activated agarose gel. The IgG (about 1mg) solution was added to the PAB variant immobilized agarose gel, and the mixture was shaken at 25°C for 30 min in pH 9.0 buffer (25 mM Tris–HCl (pH=9.0), 2.5 M NaCl, and 0.1% Tween 20). The amounts of IgG in supernatant after centrifugation were determined and the binding efficiency was calculated. The ordinate indicates the binding efficiency. [file 1754-1611-8-15-S8.pptx]
