# Supplementary material for: Engineered protein A ligands, derived from a histidine-scanning library, facilitate the affinity purification of IgG under mild acidic conditions
Source: J Biol Eng. 2014 Jul 1;8:15. doi: 10.1186/1754-1611-8-15 (PMC4107488; doi:10.1186/1754-1611-8-15)
Supplement: Additional file 6: Table S3 — PAB variants data by mutational experiments [7, 11, 21-24] and by molecular simulation calculations [25-26]. The binding affinity of IgG for PAB variants was determined using ELISA and SPR. None: Almost the same binding affinity, Very small: 2-fold to < 5-fold decrease, Small: 5-fold to < 10-fold decrease, Large: 10-fold to < 100-fold decrease, Very large: 100-fold decrease <. [file 1754-1611-8-15-S6.docx]

**Table S3. PAB variants data by mutational experiments [7, 11, 21-24] and by molecular simulation calculations [25-26].**

The binding affinity of IgG for PAB variants was determined using ELISA and SPR.

None: Almost the same binding affinity, Very small: 2-fold to < 5-fold decrease, Small: 5-fold to < 10-fold decrease, Large: 10-fold to < 100-fold decrease, Very large: 100-fold decrease <

| **Mutation** | **Decrease of binding affinity under neutral condition** | **Decrease of binding affinity under acidic condition** | ***∆pH*** | **Ref** |
| --- | --- | --- | --- | --- |
| **Q9A** | **None** | **-** | **-** | **7** |
| **Q9H** | **Very small** | **Very small** | **0.4** | **This study** |
| **Q9H, D36H** | **Large** | **N.D.** | **2.0** | **This study** |
| **Q10H** | **Small** | **Very small** | **-** | **7** |
| **Q10H** | **Large** | **Very large** | **0.2** | **This study** |
| **Q10H, N11H** | **None** | **Very large** | **1.4** | **11** |
| **Q10H, D36H** | **Very large** | **N.D.** | **3.3** | **This study** |
| **N11A** | **None** | **-** | **-** | **7** |
| **N11H** | **None** | **None** | **-** | **7** |
| **N11H, L17H** | **Large** | **N.D.** | **0.6** | **11** |
| **F13H** | **Very large** | **-** | **-** | **7** |
| **Y14E** | **Large** | **-** | **-** | **21** |
| **Y14F** | **Small** | **-** | **-** | **7, 21** |
| **Y14H** | **Small** | **-** | **-** | **21** |
| **Y14K** | **Large** | **-** | **-** | **21** |
| **Y14R** | **Small** | **-** | **-** | **21** |
| **Y14S** | **Small** | **-** | **-** | **21** |
| **E15H, L17H** | **Small** | **Large** | **1.4** | **11** |
| **L17D** | **Small** | **-** | **-** | **22、23、24** |
| **L17H** | **Small** | **Large** | **1.25** | **7** |
| **R27H** | **None** | **None** | **-** | **This study** |
| **R27H, D36H** | **Large** | **Very large** | **1.2** | **This study** |
| **N28A** | **Very small** | **-** | **-** | **22、23、24** |
| **N28H** | **None** | **Small** | **-** | **7** |
| **F30A** | **None** | **-** | **-** | **22、23、24** |
| **I31A** | **Very large** | **-** | **-** | **22、23、24** |
| **Q32H** | **None** | **Large** | **0.7** | **This study** |
| **K35A** | **Large** | **-** | **-** | **22、23、24** |
| **K35H** | **None** | **None** | **-** | **7** |
| **K35H, D36H** | **Very large** | **Very large** | **0.9** | **This study** |
| **D36H** | **Small** | **Very large** | **1.1** | **This study** |
| **Simulation** | | | | |
|  | **N11, F13, Y14, L17, E24, I31 and K35 are hot spots.** | | **-** | **25** |
|  | **F13, Y14, H18, E24, R27, and K35 are hot spots.** | | **-** | **26** |
