# Supplementary material for: Engineered protein A ligands, derived from a histidine-scanning library, facilitate the affinity purification of IgG under mild acidic conditions
Source: J Biol Eng. 2014 Jul 1;8:15. doi: 10.1186/1754-1611-8-15 (PMC4107488; doi:10.1186/1754-1611-8-15)
Supplement: Additional file 9: Table S4 — Amino acid residues of therapeutic antibodies related to the effects of D36H. In this study, Arg2519 of IgG-Fab was found to be related to the effects of D36H mutation. Of top 10 therapeutic antibodies in 2010, 80% of the amino acid residues corresponding to Arg2519 are positively charged residues. Sales rank of therapeutic antibodies in 2010 are cited from the article (John G. Elvin et al. International Journal of Pharmaceutics 440 (2013) 83– 98). [file 1754-1611-8-15-S9.docx]

**Table S4. Amino acid residues of therapeutic antibodies related to the effects of D36H.**

In this study, Arg2519 of IgG-Fab was found to be related to the effects of D36H mutation. Of top 10 therapeutic antibodies in 2010, 80 % of the amino acid residues corresponding to Arg2519 are positively charged residues. Sales rank of therapeutic antibodies in 2010 are cited from the article (John G. Elvin *et al.* International Journal of Pharmaceutics 440 (2013) 83– 98).

| **Name of a therapeutic antibody** | **Antibody** | **Amino acid residue** |
| --- | --- | --- |
| **Avastin** | **Bevacizumab** | **R** |
| **MabThera/Rituxan** | **Rituximab** | **K** |
| **Humira** | **Adalimumab** | **R** |
| **Remicade** | **Infliximab** | **K** |
| **Herceptin** | **Trastuzumab** | **R** |
| **Lucentis** | **Ranibizumab** | **R** |
| **Erbitux** | **Cetuximab** | **S** |
| **Tysabri** | **Natalizumab** | **K** |
| **Synagis** | **Palivizumab** | **T** |
| **Xolair** | **Omalizumab** | **R** |
