# Supplementary material for: Engineered protein A ligands, derived from a histidine-scanning library, facilitate the affinity purification of IgG under mild acidic conditions
Source: J Biol Eng. 2014 Jul 1;8:15. doi: 10.1186/1754-1611-8-15 (PMC4107488; doi:10.1186/1754-1611-8-15)
Supplement: Additional file 10: Figure S7 — Elution profile of IgG on affinity columns with immobilized 4×PAZ variants. (a) Elution profile of IgG from affinity columns prepared with immobilized 4×PAZ01 or 4×PAZ03. IgG was bound with pH 7.5 buffer (25 mM Tris–HCl (pH=7.5), 150 mM NaCl, and 0.1% Tween 20) at a flow rate of 0.5 mL/min, (b) Elution profile of IgG from affinity columns prepared with immobilized 4×PAZ01 or 4×PAZ03. IgG was bound with pH 7.5 buffer (25 mM Tris–HCl (pH=7.5), 150 mM NaCl, and 0.1% Tween 20) at a flow rate of 0.1 mL/min, (c) Elution profile of IgG from affinity columns prepared with immobilized 4×PAZ01 or 4×PAZ03. IgG was bound with pH 9.0 buffer (25 mM Tris–HCl (pH=9.0), 2.5 M NaCl, and 0.1% Tween 20) at a flow rate of 0.1 mL/min. Affinity columns were prepared using the 4×PAZ variants. The captured IgG on the column was eluted using a buffer with the pH indicated in the Figure. Afterwards, the residual IgG was eluted with a decreasing pH gradient. The ordinate on the right indicates the pH of the solution. The ordinate on the left indicates the absorbance at 280 nm (mAU: milliabsorbance units). The abscissa indicates the elution volume. The solid and dashed lines show the elution patterns of IgG on 4×PAZ variant columns, as shown in the Figure, and the pH value of the elution buffer, respectively. The inlets show the elution patterns with magnified scale from 0 to 8 mL. [file 1754-1611-8-15-S10.pptx]

## Slide 1
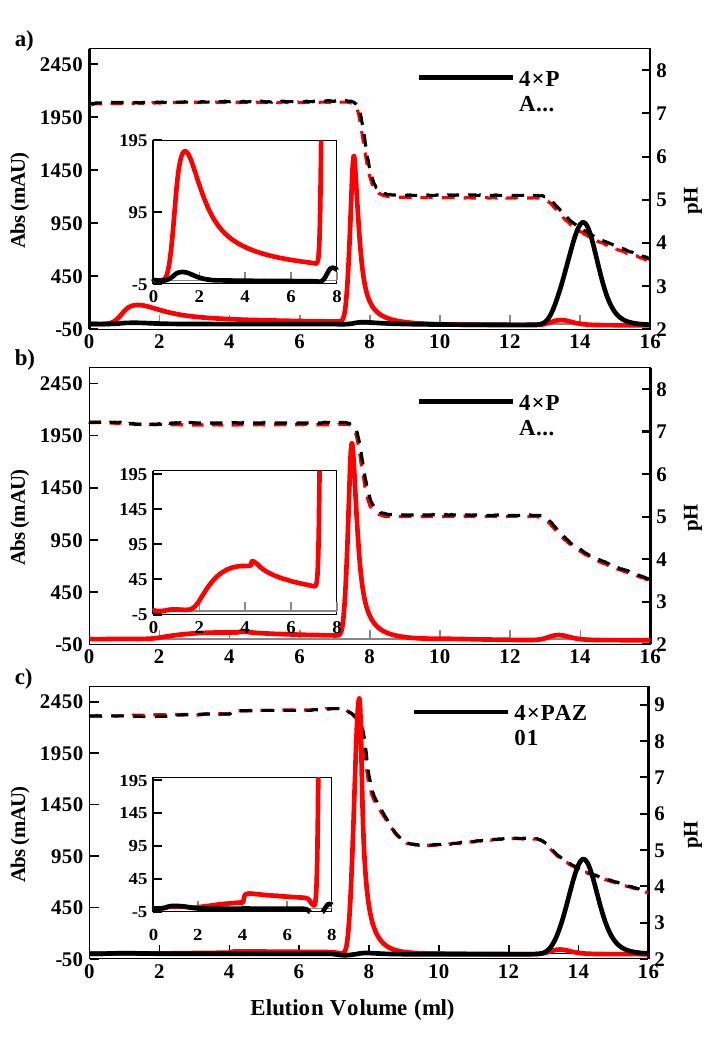

a)
### Chart
| Category | | | | |
|---|---|---|---|---|
### Chart
| Category | | |
|---|---|---|b)
### Chart
| Category | | | | |
|---|---|---|---|---|
### Chart
| Category | | |
|---|---|---|c)
### Chart
| Category | | | | |
|---|---|---|---|---|
### Chart
| Category | | |
|---|---|---|
