# Supplementary material for: Engineered protein A ligands, derived from a histidine-scanning library, facilitate the affinity purification of IgG under mild acidic conditions
Source: J Biol Eng. 2014 Jul 1;8:15. doi: 10.1186/1754-1611-8-15 (PMC4107488; doi:10.1186/1754-1611-8-15)
Supplement: Additional file 11: Table S1 — List of primers. Primers 1~4 indicate the oligonucleotides for constructing a histidine-scanning library. Primers 5~12 indicate the oligonucleotides for introducing histidine-mutations. The following abbreviations are used for mixed bases: R=(A or G), Y=(C or T), M=(A or C), K=(G or T), S=(G or C) and W=(A or T). [file 1754-1611-8-15-S11.docx]

**Table S1. List of primers.**

Primers 1~4 indicate the oligonucleotides for constructing a histidine-scanning library. Primers 5~12 indicate the oligonucleotides for introducing histidine-mutations. The following abbreviations are used for mixed bases: R=(A or G), Y=(C or T), M=(A or C), K=(G or T), S=(G or C) and W=(A or T).

| Name | Sequence | Length (nt) |
| --- | --- | --- |
| Primer 1 | CCGAATTCCGGCGGTGGAGGCTCCATGGCTGATAACAAA | 39 |
| Primer 2 | GTTTAAGTTCGGCAAATGMWGGATWTSATRRWRAGCRTKWTGWTGTTCTTTRTKAWRTTTGTTATCAGCCATGGAGCCTCC | 81 |
| Primer 3 | CATTTGCCGAACTTAAACSAWSAWCAACRYMAYGGCTTCMWTCAWAGCCTGMAWSATGACCCAAGCCAAA GCGCTAAC | 78 |
| Primer 4 | CGCAAGCTTGTCTTATTTTGGTGCTTGTGCATCATTTAGCTTTTTAGCTTCTGCTAAAAGGTTAGCGCTT TGGCTTGGGTC | 81 |
| Primer 5 | GATAACAAATTTAACAAAGAACATCAAAACGCTTTCTATGAAATC | 45 |
| Primer 6 | GATTTCATAGAAAGCGTTTTGATGTTCTTTGTTAAATTTGTTATC | 45 |
| Primer 7 | AACAAATTTAACAAAGAACAACATAATGCTTTCTATGAAATCTTAC | 46 |
| Primer 8 | GTAAGATTTCATAGAAAGCATTATGTTGTTCTTTGTTAAATTTGTT | 46 |
| Primer 9 | CCGAACTTAAACGAAGAACAACATAACGGCTTCATTCAGAGC | 42 |
| Primer 10 | GCTCTGAATGAAGCCGTTATGTTGTTCTTCGTTTAAGTTCGG | 42 |
| Primer 11 | CAACGCAACGGCTTCATTCATAGCTTAAAAGATGACCCA | 39 |
| Primer 12 | CTTCATTCAGAGCTTAAAACATGACCCAAGCCAAAGCGCTA | 41 |
